# Supplementary material for: Improving the Efficiency of Precise Genome Editing with CRISPR/Cas9 to Generate Goats Overexpressing Human Butyrylcholinesterase
Source: Cells. 2023 Jul 10;12(14):1818. doi: 10.3390/cells12141818 (PMC10378061; doi:10.3390/cells12141818)
Supplement: Supplementary file 1 [file cells-12-01818-s001.zip › cells-2404474-supplementary.pdf]

cells

MDPI

Supplementary materials

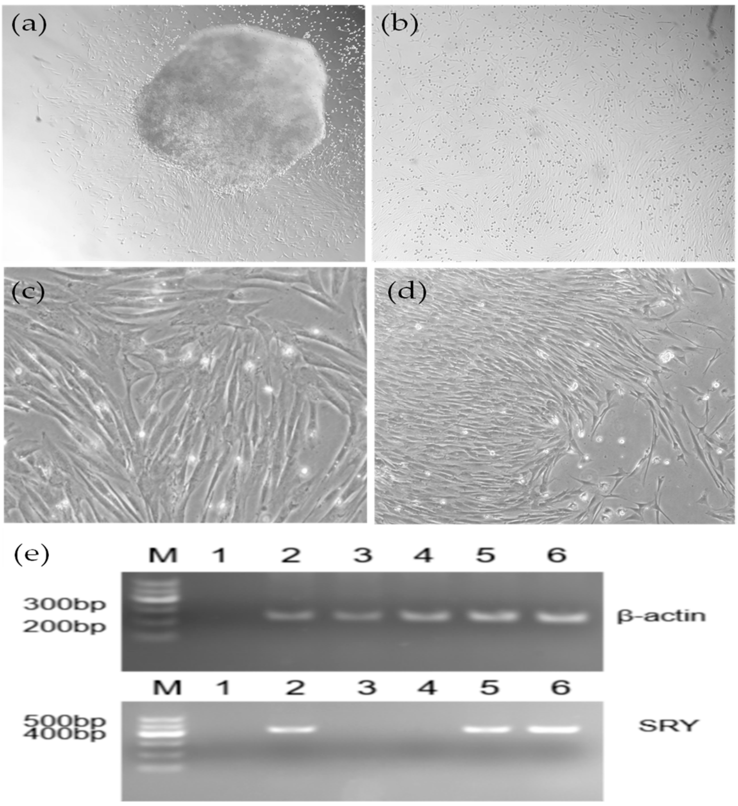

Figure S1. Construction of GFF cell lines by organization to adherent culture. (a–d) FFCs crawled out of fetal tissue after 4 days, 7 days, 10 days, and 14 days; (e) PCR amplification of *β-actin* and *SRY* gene. M, marker; 1, blank control; 2, known male goat control; 3, known female goat control; 4, fetus 1; 5, fetus 2; 6, fetus 3.

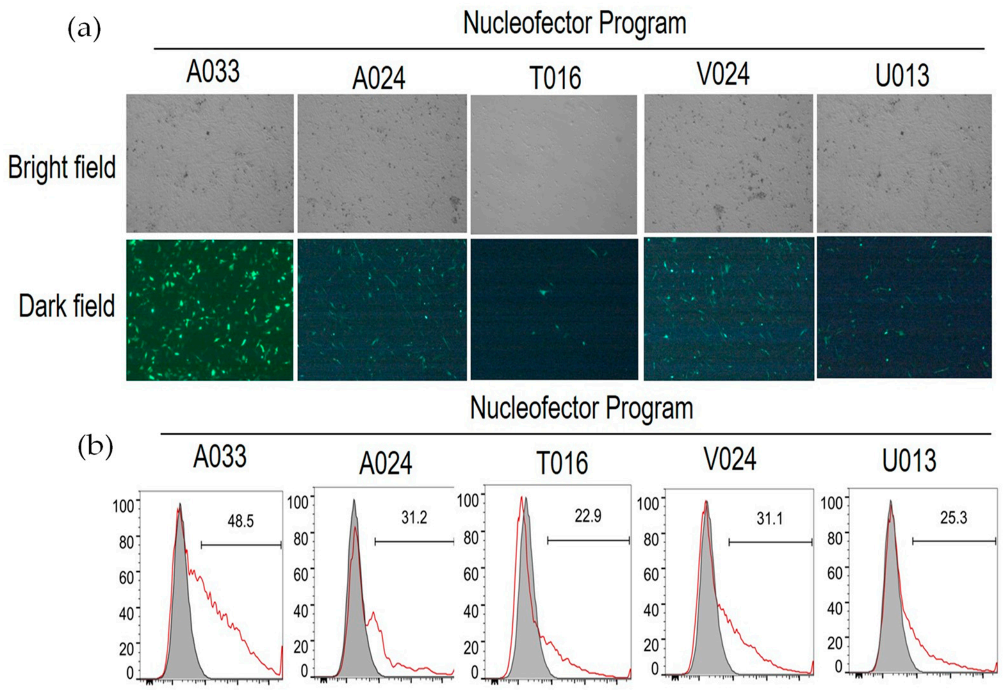

Figure S2. Effect of PX458-*FGF5* plasmid expressed in GFFs. (a) Fluorescence expression in GFFs transfected with the PX458 plasmid between different nucleofection programs at 48 h post transfection (100×); (b) Determination of the efficiencies of nucleofection by flow cytometry at 48 h post transfection. The percentages of EGFP+ cells represent the nucleofection efficiencies.

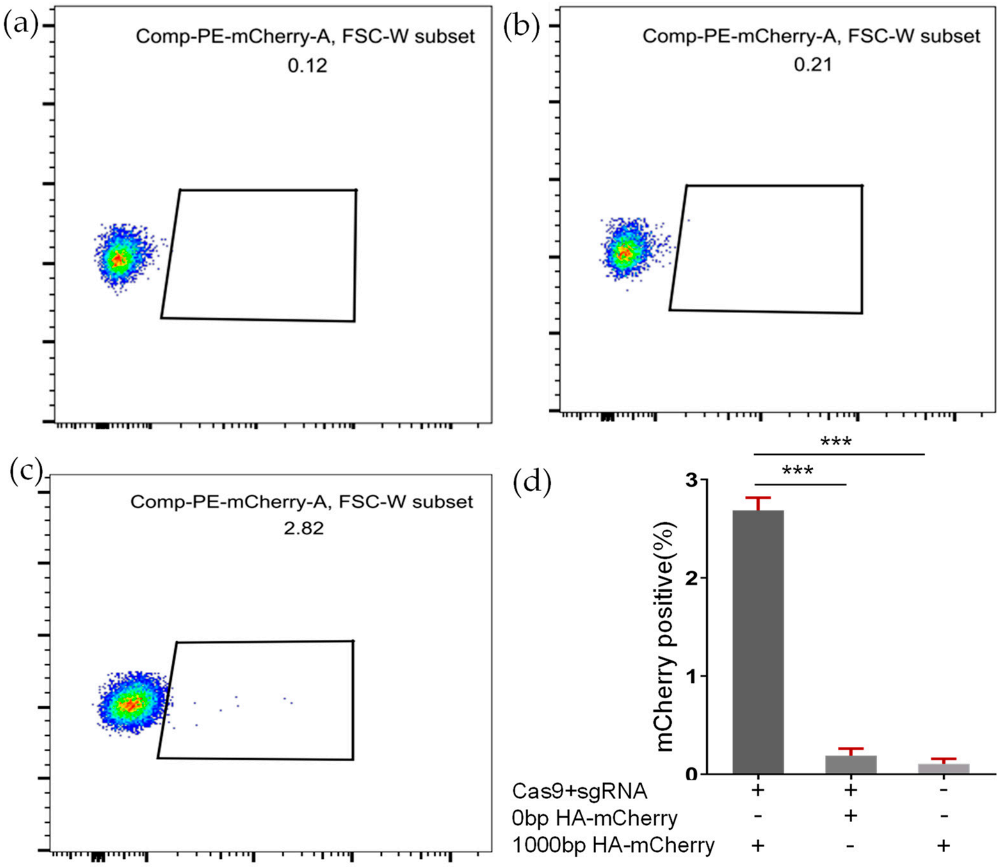

Figure S3. Detection of knock-in efficiency in GFFs. (a-c) HDR efficiency of mCherry+ knock-in of GFFs with Cas9+0bp HA-mCherry template, only 1000bp HA-mCherry template and Cas9+1000bp HA-mCherry template; (d) HDR efficiency with different control groups. n = 3 biological replicates; ***$p < 0.001$.

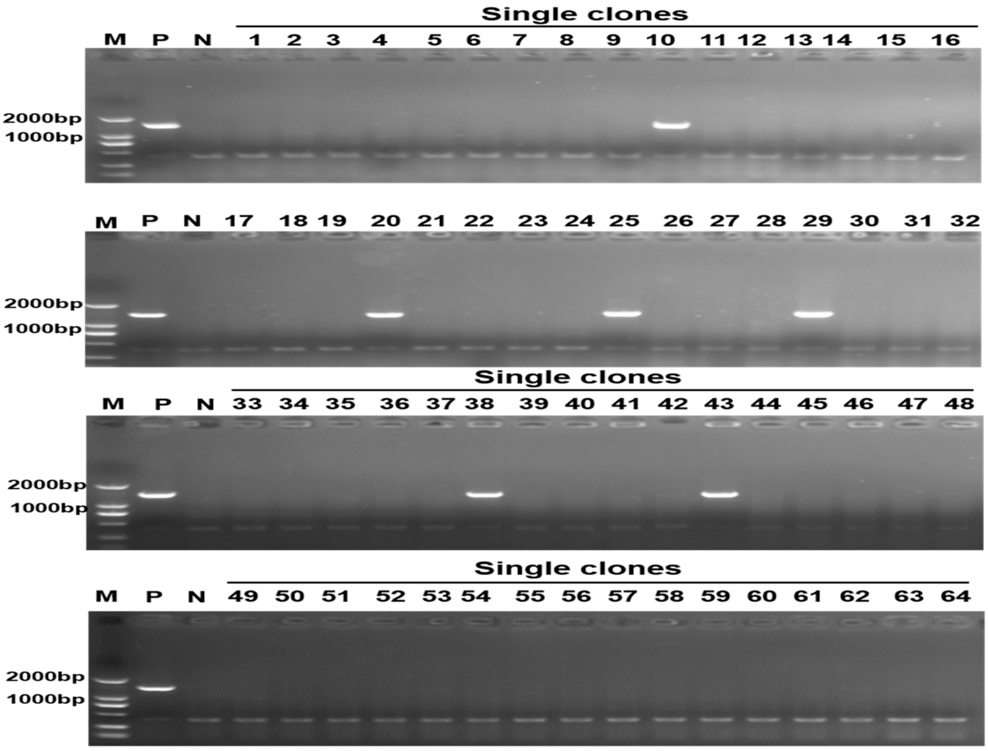

Figure S4. Positive monoclonal screening and identification of 5′ junction PCR analyses confirming the site-specific targeting in the GFF clones expressing rhBChE via primer LI-F/R. P, positive mix cell clone; N, negative wild-type clone; 1–64, monoclonal number.

Table S1. Primers for SRY identification.

Genes		Primer sequence(5′-3′)
SRY-goat	Forward:	CGAAAGGTGGGCTCTAGAGAA
	Reverse:	ATAGCTAGTAGTCTCTGTGCCT
ACTB	Forward:	CCTCCTTTCTTGCCCTTCTTT
	Reverse:	CTCGGTTGGCTTTTGTCACAC

Table S2. Primers for identification of the goat fibroblast cell line expressing rhBChE.

Primers:	Primer sequence(5′-3′)	Length
ID-F	atttcaagaaaacagctataat	3004bp/
ID-R	gaagttgccttcagagcact	394bp
RI-F	ccgcgactctagatcataat	1655bp
RI-R	ggcagatctattcactgagg	
LI-F	cctctcatcacctgatcact	1482bp
LI-R	ggctatgaactaatgaccccgt	
